# *In vitro* and *in vivo* characterization of [^64^Cu][Cu(elesclomol)] as a novel theranostic agent for hypoxic solid tumors

**DOI:** 10.1007/s00259-023-06310-4

**Published:** 2023-06-29

**Authors:** Tengzhi Liu, Maria Aanesland Dahle, Mathilde Hirsum Lystad, Laure Marignol, Morten Karlsen, Kathrine Røe Redalen

**Affiliations:** 1 Department of Physics, Norwegian University of Science and Technology, Trondheim, Norway; 2 Department of Radiology and Nuclear Medicine, St. Olavs Hospital, Trondheim University Hospital, Trondheim, Norway; 3 Applied Radiation Therapy Trinity, Discipline of Radiation Therapy, Trinity St. James’s Cancer Institute, Trinity College, Dublin, Ireland

**Keywords:** ^64^Cu-elesclomol, Hypoxia, Positron emission tomography, Theranostics, Cancer

## Abstract

**Purpose:**

Hypoxic tumors are associated with therapy resistance and poor cancer prognosis, but methods to detect and counter tumor hypoxia remain insufficient. Our purpose was to investigate ^64^Cu(II)-elesclomol ([^64^Cu][Cu(ES)]) as a novel theranostic agent for hypoxic tumors, by implementing an improved production method and assessing its therapeutic and diagnostic potential compared to the established Cu-64 radiopharmaceuticals [^64^Cu]CuCl_2_ and [diacetyl-bis(N4-methylthiosemicarbazone) [^64^Cu][Cu(ATSM)].

**Methods:**

Cu-64 was produced using a biomedical cyclotron at 12 MeV with the reaction ^64^Ni(p,n)^64^Cu, followed by synthesis of [^64^Cu]CuCl_2_, [^64^Cu][Cu(ATSM)], and [^64^Cu][Cu(ES)]. In vitro therapeutic effects were assessed in both normoxic and hypoxic cells (22Rv1 and PC3 prostate cancer cells, and U-87MG glioblastoma cells) using the clonogenic assay and analyzing cellular uptake and internalization. In vivo therapeutic effects were assessed in 22Rv1 xenografts in BALB/cAnN-Foxn1nu/nu/Rj mice receiving a single or multiple doses of radiopharmaceutical, before their feasibility to detect tumor hypoxia was assessed by positron emission tomography (PET) in 22Rv1 and U-87MG xenografts.

**Results:**

In vitro and in vivo studies demonstrated that [^64^Cu][Cu(ES)] reduced cell survival and inhibited tumor growth more effectively than [^64^Cu][Cu(ATSM)] and [^64^Cu]CuCl_2_. Hypoxia increased the cellular uptake and internalization of [^64^Cu][Cu(ES)] and [^64^Cu][Cu(ATSM)]. [^64^Cu][Cu(ES)]-PET tumor hypoxia detection was feasible and also revealed an unexpected finding of uptake in the brain.

**Conclusion:**

To the best of our knowledge, this is the first time that ES is radiolabeled with [^64^Cu]CuCl_2_ to [^64^Cu][Cu(ES)]. We demonstrated superior therapeutic effects of [^64^Cu][Cu(ES)] compared to [^64^Cu][Cu(ATSM)] and [^64^Cu]CuCl_2_ and that [^64^Cu][Cu(ES)]-PET is feasible. [^64^Cu][Cu(ES)] is a promising theranostic agent for hypoxic solid tumors.

**Supplementary information:**

The online version contains supplementary material available at 10.1007/s00259-023-06310-4.

## Introduction

The idea behind theranostics is to use one single agent for both diagnostic and therapeutic purposes. This is particularly relevant in nuclear medicine using diagnostic imaging modalities like positron emission tomography (PET) or single-photon emission computed tomography (SPECT). In PET and SPECT, a radioactive molecule is injected that will accumulate in the region the molecule is engineered to target. By detecting the radioactivity using a scanner, the presence and distribution of the radioactive molecule in the target can be imaged and quantified. For cancer, the target commonly represents the malignancy as a whole or a critical part of the malignancy. By exposing the target with short-range, therapeutic high dose radiation, the malignancy can be eliminated, while the treatment effect can be monitored with PET/computed tomography (CT) or PET/magnetic resonance imaging (MRI). Cancer theranostic is therefore a highly specific and promising diagnostic and therapeutic strategy that can enable personalized cancer treatment [1].

Tumor hypoxia describes the adverse condition within the tumor microenvironment (TME) where the tissue oxygenation is deprived as a result of the imbalance between supply and consumption, usually due to immature and chaotic development of the tumor vasculature [2]. It is well known that tumor hypoxia is associated with poor prognosis, due to resistance to chemo- and/or radiotherapy, disease progression, metastatic development, and higher probability of recurrence [3–5]. However, the assessment of tumor hypoxia remains challenging. Conventional invasive methods, though being highly sensitive and accurate within the tissue that is sampled, lack general representation of the tumor microenvironment and are limited by accessibility of the tumor [6]. Non-invasive methods, in particular PET, has emerged as a more sensitive and representative method for hypoxia detection. The copper-based hypoxia PET tracer [^64^Cu][Cu-diacetyl-bis(N4-methylthiosemicarbazone)] ([^64^Cu][Cu(ATSM)]) has demonstrated favorable potentials compared to the fluorine-based tracers such as [^18^F]fluoromisonidazole ([^18^F]F-MISO) or [^18^F]fluoroazomycin arabinoside ([^18^F]F-FAZA). Although [^18^F]F-MISO is shown to detect tissue oxygenation, it suffers from slow uptake and clearance (images usually taken at least 4 h after injection), leading to poor image quality, questionable representations due to the dynamic nature of hypoxia, in addition to lengthy examination time. On the other hand, [^18^F]F-FAZA is significantly more hydrophilic and suffers from poor penetration of the blood–brain-barrier as well as high urinary bladder uptake [7–9]. However, the tracer uptake mechanism remains controversial, having a similar in vivo distribution as ionic copper [7, 10, 11]. Although prior studies have demonstrated promising results from the use of [^64^Cu]CuCl_2_ and [^64^Cu][Cu(ATSM)] as theranostic agents, newer copper-based candidates are being investigated in order to optimize both the diagnostic and therapeutic potential for targeting aggressive, hypoxic tumor cells [12–18].

Elesclomol (ES) is a chemotherapeutic drug that has been evaluated clinically for metastatic melanoma. ES targets the mitochondria of cancer cells with elevated oxidative stress, where it subsequently induces apoptosis [19–22]. The efficacy of ES is rationalized by chelation with copper outside of the cells and entered as Cu(II)-ES (Cu(ES)), where Cu(II) is reduced to Cu(I) while reactive oxygen species (ROS) are generated [18]. This mechanism presents an opportunity to combine ES with Cu-64 and use [^64^Cu][Cu(ES)] as a theranostic agent for hypoxic tumors, especially for regions with over reduced intracellular state as a result of hypoxia [23]. In addition to being a positron emitter, Cu-64 also emits low energy, high linear energy transfer (LET) Auger electrons, which have demonstrated to induce tumor cell death with high efficiency [24, 25]. With [^64^Cu][Cu(ES)], Cu-64 is delivered into the tumor mitochondria by the help of ES in order to induce the high-LET therapeutic radiation effect, while the chemical concentration of ES remains orders of magnitudes below toxic level.

In this study, our first aim was to implement a production method of [^64^Cu]CuCl_2_ and subsequently label ATSM and ES in high molar activity. ES has not been labeled with [^64^Cu]CuCl_2_ before. Secondly, we aimed to investigate the therapeutic effects of [^64^Cu][Cu(ES)] as compared to [^64^Cu]CuCl_2_ and [^64^Cu][Cu(ATSM)], both in vitro using cell cultures from prostate cancer and glioblastoma and in vivo in prostate cancer xenografts receiving single- and multiple doses of [^64^Cu][Cu(ES)] in comparison to [^64^Cu][Cu(ATSM)]. Third, the potential of [^64^Cu][Cu(ES)] to detect tumor hypoxia compared to [^64^Cu]CuCl_2_ and [^64^Cu][Cu(ATSM)] was evaluated with PET/MR imaging of both prostate and glioblastoma xenografts.

## Materials and methods

### Production of Cu-64 and radiochemical synthesis

Synthesis of ATSM and ES precursors and radiosynthesis of [^64^Cu]CuCl_2_, [^64^Cu][Cu(ATSM)], and [^64^Cu][Cu(ES)] are described in the Supplementary Information. Production of Cu-64 was performed at the PET Center at St. Olavs Hospital using a modified TBP/TK201 column method as previously reported [26]. Cu-64 was produced on a GE PETrace 800 series biomedical cyclotron following the ^64^Ni(p,n)^64^Cu nuclear reaction at 12 MeV proton energy, and synthesized into [^64^Cu]CuCl_2_, [^64^Cu][Cu(ATSM)], and [^64^Cu][Cu(ES)]. Before bombarding the Ni-64 target with protons, the target was electroplated with Ni-64 by electrodeposition of a ammonium chloride buffered solution of nickel nitrate (^64^Ni(NO_3_)_2_). Typical irradiation parameters were 35 µA, for 3 h on a 50 mg target. Electrodeposition of Ni-64 on the target was performed on a Comecer ALCEO solid targeting processing system, as previously described [27].

### Cell cultures

The human prostate cancer cell line 22Rv1 (ATCC, Manassas, VA, USA) and PC3 (ATCC) was maintained in RPMI1640 medium (Sigma-Aldrich) with fetal bovine serum (10%, Sigma-Aldrich) and penicillin (0.1 μg/mL, Sigma-Aldrich) at 37 °C, 5% CO_2_ and maintained in exponential growth. The human glioblastoma cancer cell line U-87MG (ATCC) was maintained in Eagle’s minimum essential medium (ATCC) with fetal bovine serum (10%, Sigma-Aldrich) and penicillin (0.1 μug/mL, Sigma-Aldrich) at 37 °C, 5% CO_2_ and maintained in exponential growth.

### Experimental hypoxia

Hypoxic environment was generated using the Oxoid Anaerogen atmosphere generation system (Thermo Fisher, Waltham, MA) with a 2.5 L sealed container, an Oxoid AnaeroGen™ 2.5L Sachet (Thermo Fisher) and an Oxoid Resazurin Anaerobic Indicator (Thermo Fisher). The sealed containers were placed in the incubator at 37 °C. Hypoxic condition was achieved within 30 min after the sachet was placed into the sealed box, which maintains 8% CO_2_ and < 1% O_2_. The oxygen concentration was confirmed by the color change of the indicator, which was added alongside the sachet. Under normoxic conditions, the indicator turns red, while in hypoxia (< 1% O_2_), the indicator turns white.

Methodological description of preparation of cell lysates and Western blotting performed on normoxic and hypoxic cells treated with the radiopharmaceuticals can be found in Supplementary Information.

### Clonogenic assay

The clonogenic assay was performed using 22Rv1, PC3, and U-87MG cells, all exposed to [^64^Cu]CuCl_2_, [^64^Cu][Cu(ATSM)], and [^64^Cu][Cu(ES)] at activities of 0, 2, 4, 8, 32, 64, and 128 Bq/cell in normoxic and hypoxic conditions. The clonogenic assay was performed following a modified protocol from Franken et al. [28]. Briefly, a suspension of cells containing 400 cells in 2.97 mL of growth medium was added to each well (Corning Costar 6-well TC-treated well plates, Corning, NY, USA) 24 h before treatment. The well plates were incubated at 37 °C, 5% CO_2_ for 24 h prior to treatments. For hypoxic samples, the plates were placed in hypoxic conditions (37 °C, < 1% O_2_, 8% CO_2_) 4 h prior to treatment, while the normoxic samples remained in normoxic conditions (37 °C, 5% CO_2_). During treatment, 30 μL of radiopharmaceuticals at different activity concentrations or vehicles was added to each well. After the treatment, the hypoxic samples were placed in a freshly generated hypoxic environment for 4 h before returning to normoxic conditions. Normoxic samples were placed in the incubator in normoxic conditions directly. After 14 days, the plates were removed from the incubator and the growth medium was carefully aspirated. The plates were allowed to dry in air, before 5 mL of ethanol (75%) was added to each well and left for 15 min at room temperature. The ethanol was aspirated from each well and the plates were dried in air for 5 min. Crystal violet (3 mL, 1% in absolute ethanol) was added to each well with closed lid and kept at room temperature for 30 min. The crystal violet was then removed, and the plates were carefully rinsed in water and dried. The colonies were counted to calculate the plating efficiency and therapeutic effects of the different treatments. Experiments were performed in three technical and three biological replicates. After counting the number of colonies, the average number per biological replicate was used to calculate the plating efficiency (PE): PE% = (average number of colonies per biological replicate/number of cells seeded) × 100%. The survival fraction (SF) was calculated by the ratio of PE in treated cells and PE in the control group: SF% = (PE of treated sample/PE of control sample) × 100%. The mean and standard deviation of SF were calculated based on three biological replicates.

### *In vivo* characterization in mouse xenografts

All animal experiments were conducted using male BALB/cAnN-Foxn1nu/nu/Rj nude mice (Janvier labs, Pays de la Loire, France) purchased at 6 weeks of age. The mice were housed in individually ventilated cages with groups of five mice/cage and with 12 h day/night cycles. Free access to food and water was provided, as well as enrichment elements including paper housing and nesting materials. All experiments with animals were approved by the Norwegian Food Safety Authority, and the reporting is according to the ARRIVE 2.0 guidelines.

Mice were anaesthetized with 2–3% isoflurane prior to tumor inoculation. A volume of tumor cell suspension containing 0.5 million 22Rv1 or U-87MG cells (100 μL) was injected subcutaneously on the lateral side of the left hind leg. The tumor cell suspension was prepared by mixing 5 mL of cells in growth medium (10*10^6^ cells/mL) with 5 mL of Matrigel (Corning Life Science, Tewksbury, MA, USA) at 37 °C. The shortest (W) and longest (L) perpendicular tumor diameters were measured twice a week using a caliper. The tumor volume (V) was calculated by V = π/6*L*W^2. Body weights of mice were monitored twice a week.

### Dynamic PET/MR imaging

PET/MR images were obtained using a Bruker Biospec 70/20 USR Avance III 7 Tesla small animal MRI scanner with micro-PET insert (Bruker Corporation, Billerica, MA, USA). Acquisition of PET was performed continuously for 90 min after tracer injection. MRI was acquired simultaneously with PET. T2-weighted images (echo time = 1.5 ms, repetition time = 4 ms, slice thickness = 0.5 mm) were acquired in the coronal direction to provide anatomical images for PET/MR colocalization and tumor identification. Mice bearing 22Rv1 tumors at approximately 10–15 mm in diameter were used for dynamic PET/MRI. To reduce the number of mice, the same mice first participated in the therapy study (described below). A BD Neoflon Pro IV 24-gauge catheter (Becton, Dickinson and Company, Franklin Lakes, NJ, USA) was inserted into the tail vein, before the mouse was transported into the scanner. Approximately 30 MBq of [^64^Cu][Cu(ATSM)] or [^64^Cu][Cu(ES)] was injected intravenously (i.v.) through the catheter immediately prior to the PET acquisition. Physiological parameters including body temperature and pulse rate were monitored during the scan. Immediately after PET/MR acquisition, the animal was sacrificed. The tumor was dissected and preserved in formalin solution for ex vivo immunohistochemical (IHC) staining of the tumor tissue. Two hours before sacrifice of the mouse (before the start of the PET), the mice had been injected with 1.5 mg of the hypoxia marker pimonidazole in 50 μL suspension intraperitoneally. Histological analysis of tissue samples is described in Supplementary Information. Dynamic PET images were reconstructed with Paravision 360 (Bruker) using the ordered subset expectation maximization (OSEM-3D) method with a slice thickness of 0.25 mm. For PET/MR visualization, the PET images were reconstructed in 5-min intervals during the entire scan duration. Uptake analyses in images were based on images reconstructed every minute of the scan duration.

### *In vivo* therapy study and static PET/MR imaging

All mice developed tumors after inoculation of 22Rv1 cells. One mouse in the [^64^Cu][Cu(ATSM)] group died right before the therapy experiments started (due to fighting). For the in vivo therapy study, mice bearing 22Rv1 tumors were allocated to one of four groups when the shortest tumor diameter reached approximately 5 mm, as determined a priori. The weight of the mice at the start of the experiments was 24.57 (mean) ± 1.19 (s.d.) grams. The number of mice per experimental group was determined based on our previous experience with similar type of experiments. Mice in group 1 (*N* = 4) received vehicle (saline) as control group. Mice in group 2 (*N* = 9) were given a single dose (SD) of [^64^Cu][Cu(ATSM)], mice in group 3 (*N* = 5) were given a single dose of [^64^Cu][Cu(ES)], and mice in group 4 (*N* = 5) were given multiple doses (MD) consisting of three repeated doses of [^64^Cu][Cu(ES)] on day 0, day 7, and day 21. Each dose contained 25–37 MBq of activity. Due to production logistics, the tumors in group 4 were slightly larger (shortest diameter 7–8 mm) at the start of treatment. Otherwise, we randomized mice in order to have an equal distribution of tumor volumes per group. Mice were anaesthetized with 2–3% isoflurane prior to treatment, and the treatment was administered i.v. through the tail vein. After treatment, mice were returned to their home cage under observation. Tumor sizes and body weights were measured twice a week. Mice were sacrificed when the largest tumor diameter reached a humane endpoint of 15 mm, according to regulations set by the Norwegian Food Safety Authority. In vivo therapy experiments were carried out by TL, MAD, and MK, who were not blinded to the group allocation since different treatments were administered. However, tumor measurements were performed blindly to group allocation.

At the day of sacrifice, some mice bearing 22Rv1 or U-87MG tumors with 10–15 mm in length underwent static PET/MRI. Mice were anaesthetized before administration of approximately 15 MBq [^64^Cu]CuCl_2_, [^64^Cu][Cu(ATSM)], or [^64^Cu][Cu(ES)]. After 3 h, the mice were anaesthetized again and PET/MRI was conducted. PET was acquired for 10 min simultaneously with T2-weighted anatomical MRI in the coronal direction. Mice were sacrificed immediately after the imaging.

### Dosimetry

The absorbed dose was calculated from the product of the cumulated activity and S-value. The cumulated activity was measured from the image-derived time activity curve (TAC) based on PET scans at 1, 4, and 24 h time points post injection in two healthy animals given 9.37 ± 0.11 MBq of [^64^Cu][Cu(ES)]. The S-value of Cu-64 was obtained from International Commission on Radiological Protection (ICRP) Publication 107 Nuclear Decay Data for Dosimetric Calculations [29]. The data was validated by harvesting all relevant organs after 24 h and measuring them on a gamma counter. The measured counts per minute (CPM) was converted to KBq by using an external linear calibration curve and decay correcting the activity back to 1-h post-injection (Perkin Elmer, 1480 Wizard3).

### Statistical analysis

Paired (clonogenic assays, cellular uptake, and internalization) and un-paired (tumor volumes) *t*-tests were used for statistical analysis using GraphPad Prism version 8.0 for Mac (GraphPad Software, Inc., San Diego, CA). *p*-value < 0.05 was considered statistically significant.

## Results

**Fig. 1:**
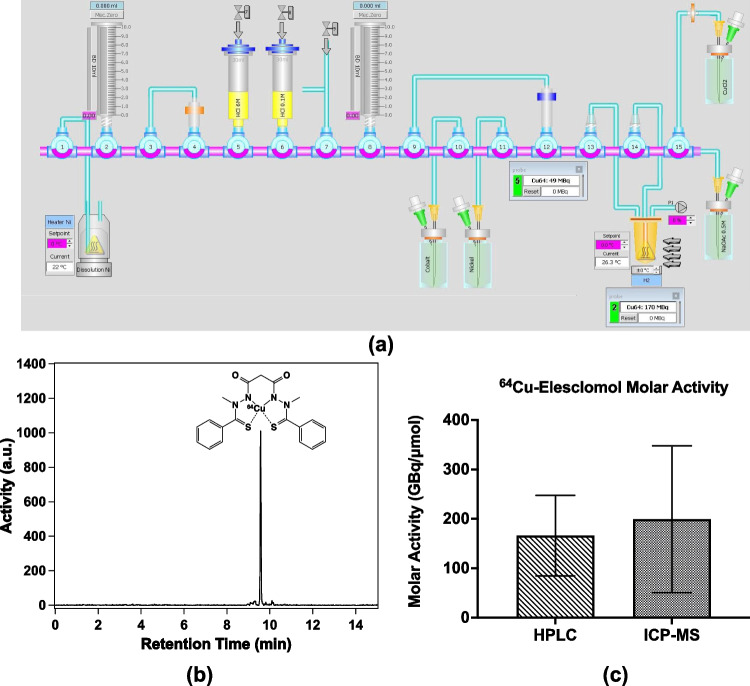
**a** Schematic of the synthesis module for the separation and purification of [^64^Cu]CuCl_2_ from unreacted Ni-64, radioactive cobalt byproducts (^55,56,57,58,61^Co). **b** High performance liquid chromatography (HPLC) of a [^64^Cu][Cu(ES)] sample analyzed with a reversed-phase C18 column and a gradient consisting of 80% solvent A (acetonitrile) and solvent B (water with 0.1% formic acid) from 20 to 100% during the first 10 min. **c** Analysis of [.^64^Cu][Cu(ES)] molar activity of the final products using HPLC (left, *N* = 6) and ICP-MS (right, *N* = 4)

The average molar activity of the buffered [^64^Cu]CuCl_2_ solution was 199 ± 148 GBq/µmol end of production (EOP) based on ICP-MS. The yield of [^64^Cu]CuCl_2_ at EOP was 1.14 ± 2.7 MBq/(μAh·mg), giving approximately 5–6 GBq [^64^Cu]CuCl_2_ in 2 mL of volume. The final pH of the solution was 4.7 ± 0.2. The radiolabeling yielded a product with radiochemical purity ≥ 98% and molar activity 166 ± 81 GBq/µmol end of synthesis (EOS) for [^64^Cu][Cu(ES)], as analyzed with HPLC (Fig. 1). The stability of [^64^Cu][Cu(ES)] was evaluated in both mouse and human serum, confirming that [^64^Cu][Cu(ES)] is stable in serum during 4 h, as shown in Supplementary Figure S1.

**Fig. 2:**
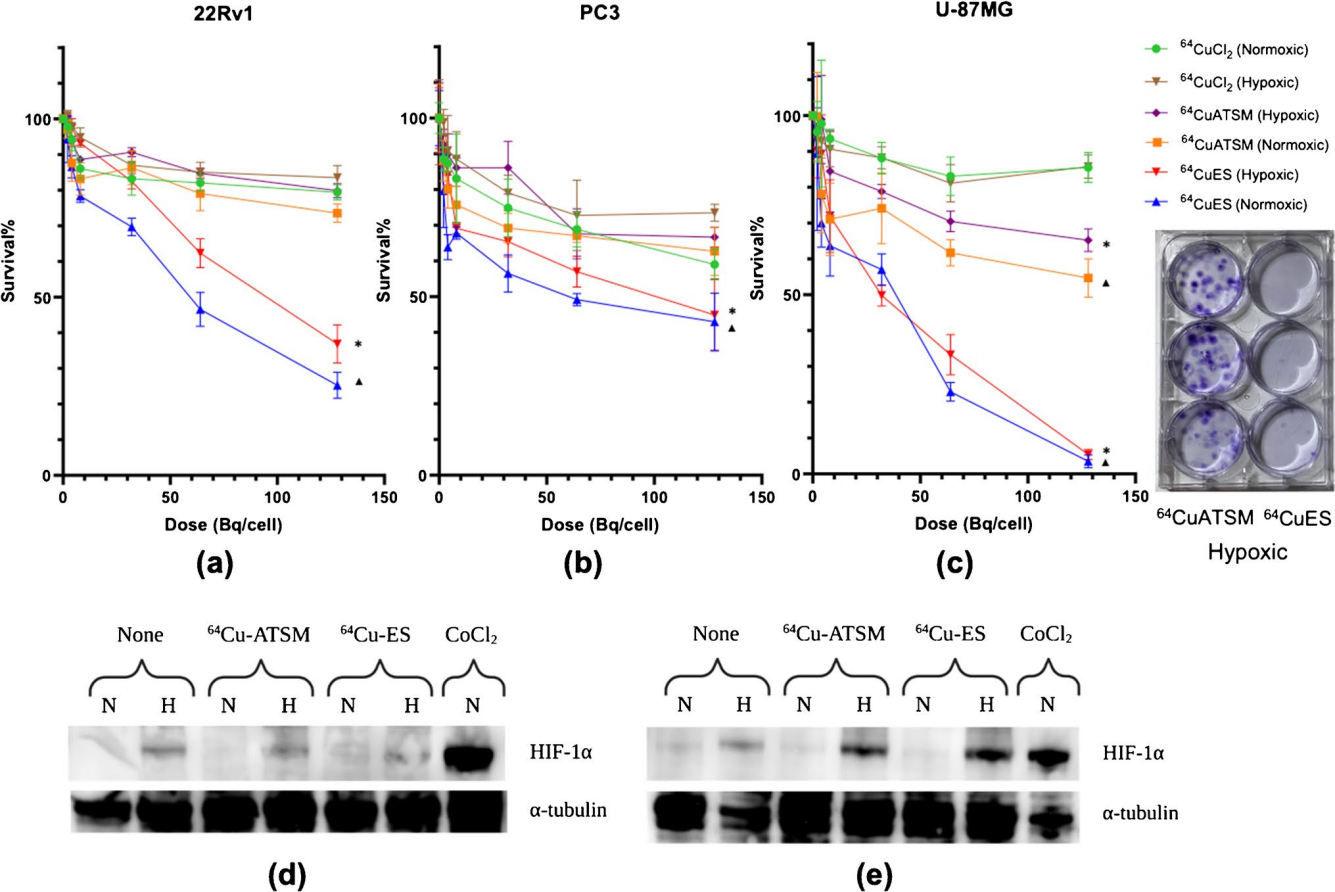
Clonogenic survival assay shows dose-dependent survivals in three human cancer cell lines (22Rv1, PC3, and U-87MG, respectively) treated with [^64^Cu]CuCl_2_, [^64^Cu][Cu(ATSM)], or [^64^Cu][Cu(ES)] in normoxic or hypoxic conditions at 0, 2, 4, 8, 32, 64, and 128 Bq/cell of activity (*N* = 3). Significant differences compared to [^64^Cu]CuCl_2_ in normoxia and hypoxia was labeled with “▲” and “*,” respectively (**a–c**)**. d** and **e** A photo is shown of a 6-well plate with stained colonies after treatment with [^64^Cu][Cu(ATSM)] (left) and [^64^Cu][Cu(ES)] (right) under hypoxic conditions. Western blot immunostaining of cell lysates treated with [^64^Cu]CuCl_2_, [^64^Cu][Cu(ATSM)], or [^64^Cu][Cu(ES)] in normoxic (N) or hypoxic (H) conditions at 4 Bq/cell confirmed the expression of HIF-1α in hypoxic samples (*N* = 3)

Treatment with [^64^Cu][Cu(ES)] resulted in reduced survival in all three cell lines (Fig. 2a–c). With an activity of 128 Bq/cell, [^64^Cu][Cu(ES)] effectively reduced the survival of U-87MG cells to 3.5% ± 1.8% (*p* = 0.003 compared to the control) in normoxia and 5.1% ± 1.4% (*p* = 0.001) in hypoxia, while treatment with [^64^Cu][Cu(ATSM)] gave a survival of 54.7% ± 5.5% (*p* = 0.009) in normoxia and 65.2% ± 3.2% (*p* = 0.172) in hypoxia. Treatment with [^64^Cu]CuCl_2_ gave only around 15% reduced survival in U-87MG cells in both normoxia and hypoxia compared to the untreated control. In the 22Rv1 and PC3 cells, the same trend as with U-87MG were seen, although the percentage of survival reduction was lower. For all three cell lines, the hypoxic cells were more resistant to treatment with [^64^Cu][Cu(ATSM)] and [^64^Cu][Cu(ES)] than the cells in normoxia, whereas the treatment with [^64^Cu]CuCl_2_ was less affected by hypoxia. The induction of hypoxia was confirmed by the expression of HIF-1α in Western blots in both treated and untreated samples (Fig. 2d, e).

**Fig. 3:**
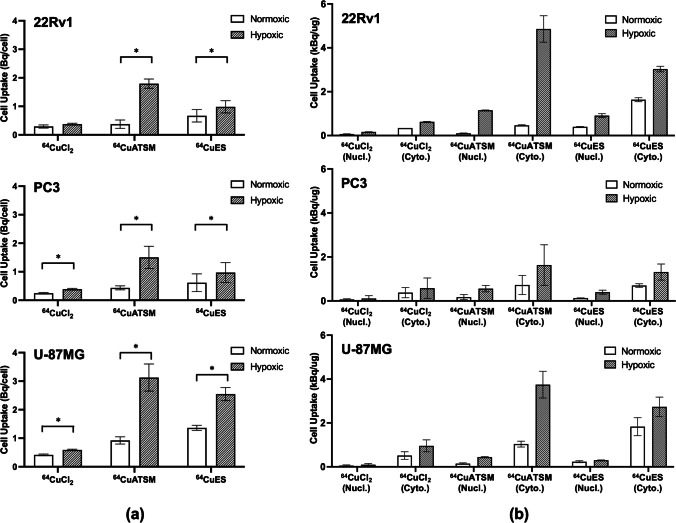
**a** Whole cell uptake of [^64^Cu]CuCl_2_, [^64^Cu][Cu(ATSM)], or [^64^Cu][Cu(ES)] in normoxic or hypoxic conditions with three human cancer cell lines (22Rv1, PC3, and U-87MG) shows significant increase of Cu-64 uptake in [^64^Cu][Cu(ATSM)] and [^64^Cu][Cu(ES)] under hypoxia (*N* = 3). Cells were given 4 Bq/cell activity. Significant difference between treatment in normoxia and hypoxia is labeled with “*.” **b** Uptake of Cu-64 in cell nucleus and cytoplasm measured in nuclear and cytoplasmic cell lysates demonstrates internalization of Cu-64 into the cell nucleus under hypoxia (*N* = 2)

Whole cell uptake studies showed that hypoxia increased the uptake of Cu-64 in all three cell lines (22Rv1, PC3, U-87MG) (Fig. 3a). For [^64^Cu]CuCl_2_, the increase was low but significant for PC3 and U-87MG (*p* = 0.008, *p* < 0.001, respectively), while for [^64^Cu][Cu(ATSM)] and [^64^Cu][Cu(ES)], the increased uptake in hypoxia was higher and significant for all three cell lines ([^64^Cu][Cu(ATSM)]: *p* = 0.014, 0.007, 0.022, respectively, and [^64^Cu][Cu(ES)]: *p* = 0.013, 0.001, 0.006, respectively). Overall, both [^64^Cu][Cu(ATSM)] and [^64^Cu][Cu(ES)] had higher uptake of Cu-64 compared to [^64^Cu]CuCl_2_ for both normoxic and hypoxic cells across all cell lines. The level of uptake was similar in 22Rv1 and PC3 cells, while the U-87MG cells had higher uptake both in normoxic and hypoxic cells.

When investigating the internalization of Cu-64, it was confirmed that most of the Cu-64 was in the cytoplasm, while some also was transferred into the cell nucleus (Fig. 3b). Compared to normoxic cells, the uptake in both the nucleus and cytoplasm increased for hypoxic cells from all three cell lines. This effect was not significant for treatment with [^64^Cu]CuCl_2_, while for both [^64^Cu][Cu(ATSM)] and [^64^Cu][Cu(ES)], the effect was significant for both the nuclear and cytoplasmic uptake, although the effect was most pronounced for [^64^Cu][Cu(ATSM)]. [^64^Cu][Cu(ES)] demonstrated a higher uptake in normoxic cells than both [^64^Cu][Cu(ATSM)] and [^64^Cu]CuCl_2_.

**Fig. 4:**
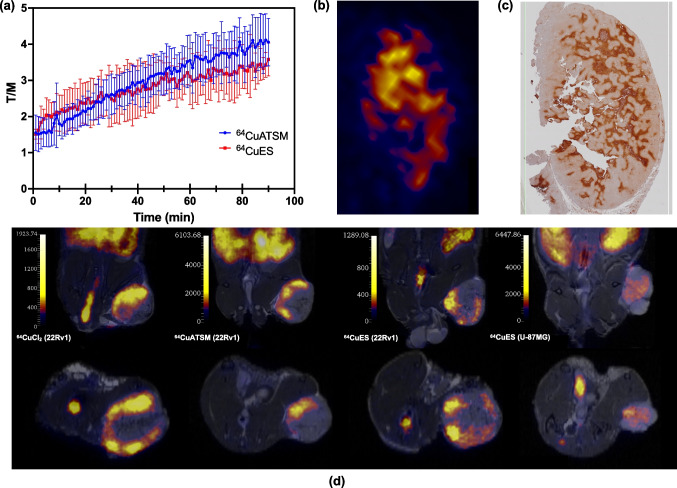
**a** Tumor-to-muscle ratio (T/M) of 90 min dynamic PET scans (1-min reconstructions) of tumor (22Rv1) bearing Bulb/c nude mice administered with [^64^Cu][Cu(ATSM)] or [^64^Cu][Cu(ES)] shows increasing uptake of the tracers during the duration of the scans (*N* = 2). **b** and **c** Slices taken from the same region of a tumor showing similar uptake regions both in **b** PET ([^64^Cu][Cu(ATSM)], 1-min reconstruction at 90 min p.i.) and in **c** Immunohistochemical (IHC) staining with anti-pimonidazole antibody. Antibody was administered i.p. 120 min prior to sacrifice of the mouse. Slice directions between PET and IHC may vary slightly. **d** PET/MR images (10-min scans) of xenograft bearing mice. From left: 22Rv1 xenograft given [^64^Cu]CuCl_2_, 22Rv1 xenograft given [^64^Cu][Cu(ATSM)], 22Rv1 xenograft given [^64^Cu][Cu(ES)], and U-87MG xenograft given [^64^Cu][Cu(ES)]. Images were taken 3 h after injection of the tracers. The unit of the scale bars is kBq/mL

Dynamic PET/MRI of 22Rv1 xenografts showed that administration of both [^64^Cu][Cu(ATSM)] and [^64^Cu][Cu(ES)] resulted in increasing accumulation of Cu-64 activity in the xenograft compared to normal tissue (muscle) (Fig. 4a). As in other studies with Cu-64, liver is the organ with the highest uptake. The increase did not reach maximum during the 90 min scan time. Hypoxia staining of excised tissue revealed colocalization of PET hotspots with hypoxic areas (Fig. 4b). In a separate experiment, static PET/MRI 3 h after injection of [^64^Cu]CuCl_2_, [^64^Cu][Cu(ATSM)], and [^64^Cu][Cu(ES)] identified similar signal intensity and uptake patterns in the 22Rv1 xenografts (Fig. 4d). The imaging experiment was also repeated with U-87MG xenografts, confirming the same effects for [^64^Cu][Cu(ATSM)] and [^64^Cu][Cu(ES)].

The absorbed doses of relevant organs from two healthy animals injected with [^64^Cu][Cu(ES)] was estimated for the following organs: kidneys (76.3 ± 17.1 mGy/MBq), liver (221.2 ± 42.4 mGy/MBq), spleen (30.5 ± 4.2 mGy/MBq), lung (79.9 ± 9.3 mGy/MBq), and brain (69.7 ± 1.74 mGy/MBq).

**Fig. 5:**
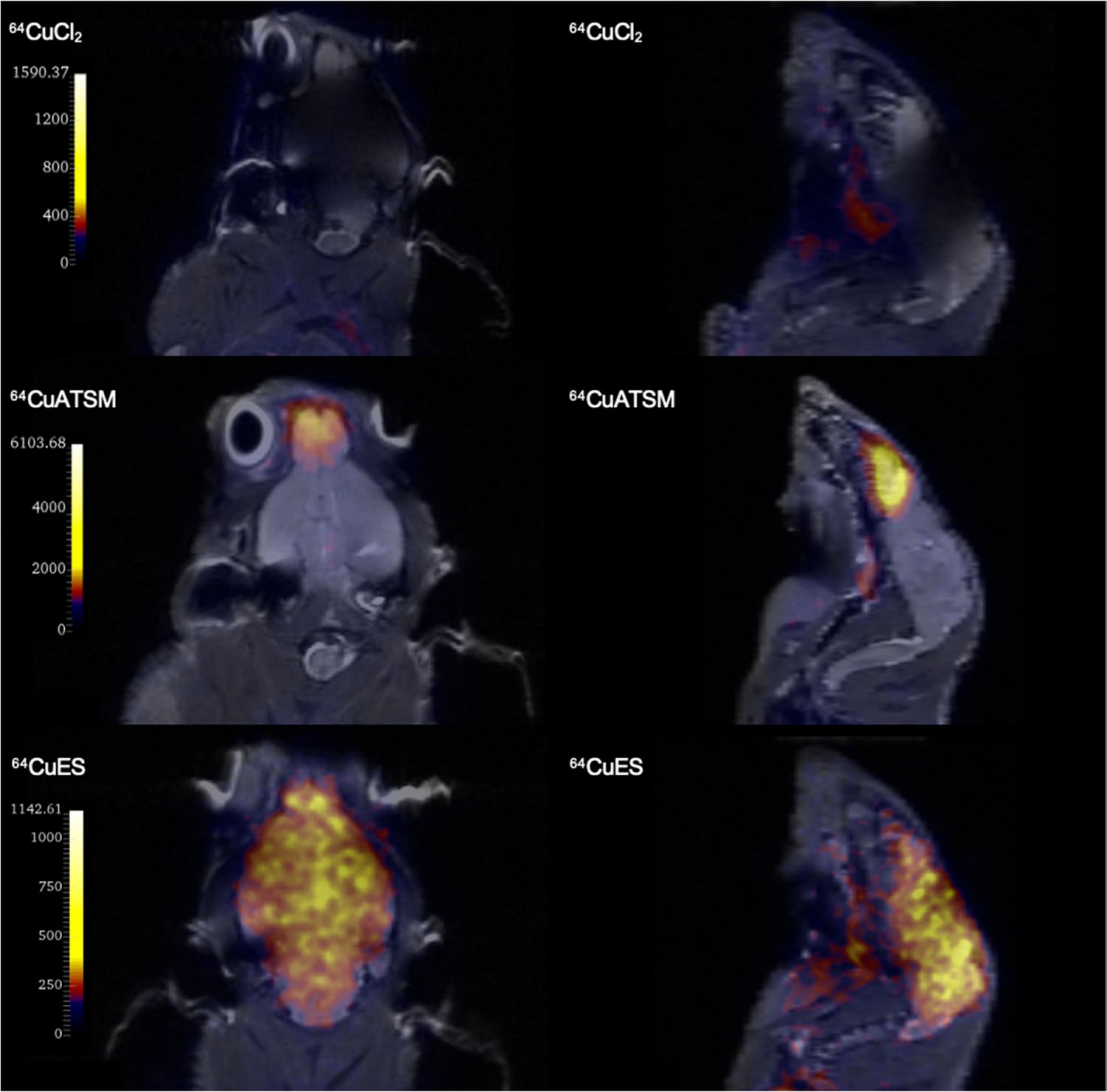
PET/MR images (10-min scans) of mouse brain uptakes 3 h post injection of [^64^Cu]CuCl_2_ (top), [^64^Cu][Cu(ATSM)] (middle), or [^64^Cu][Cu(ES)] (bottom) from coronal (left) and sagittal (right) planes. [^64^Cu]CuCl_2_ was not taken up in the brain, [^64^Cu][Cu(ATSM)] shows a hot-spot uptake in the front of the brain in the region close to the olfactory bulb, while [^64^Cu][Cu(ES)] shows significant uptake of the tracer in the whole brain. The unit of the scale bars is kBq/mL

An unexpected finding was the discovery of the differences in brain uptake after administration of [^64^Cu]CuCl_2_, [^64^Cu][Cu(ATSM)], and [^64^Cu][Cu(ES)]. [^64^Cu]CuCl_2_ did not show any significant brain uptake, [^64^Cu][Cu(ATSM)] was taken up in the frontal region of the brain, while [^64^Cu][Cu(ES)] appeared to have crossed the blood brain barrier (BBB), showing a high uptake across the whole brain (Fig. 5).

**Fig. 6:**
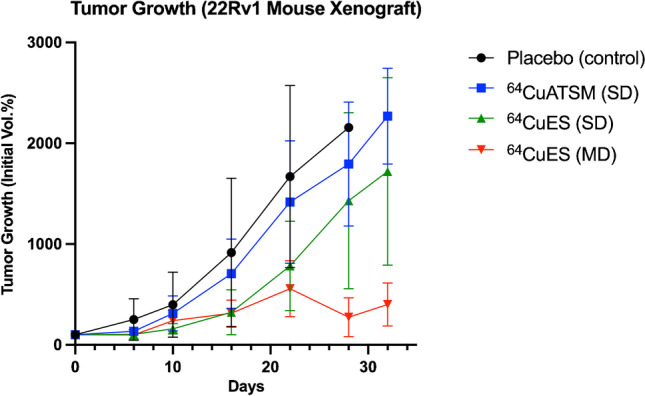
Tumor growth of 22Rv1 human prostate cancer xenografts in mice presented as percentage of the initial tumor volume, recorded from the day of first treatment (day 0). Black circles: control group, vehicle administered once on day 0 (*N* = 4); blue squares: single-dose of [^64^Cu][Cu(ATSM)] administered on day 0 (*N* = 9); green triangles: single-dose of [^64^Cu][Cu(ES)] administered on day 0 (*N* = 5); red inverted-triangles: multiple-doses of [^64^Cu][Cu(ES)] administered on days 0, 7, and 21 (*N* = 5)

When evaluating the therapeutic effects, administration of [^64^Cu][Cu(ATSM)] and [^64^Cu][Cu(ES)] both reduced the tumor growth in 22Rv1 mouse xenografts (Fig. 6). Compared to the untreated animals, a single dose of [^64^Cu][Cu(ATSM)] gave a small tumor growth inhibition. The single dose of [^64^Cu][Cu(ES)] resulted in significantly reduced tumor growth compared to the control animals. The multiple doses of [^64^Cu][Cu(ES)] gave the most significant reduction of tumor growth.

## Discussion

In this study, we implemented a method for improved production of [^64^Cu]CuCl_2_ and labeled it, for the first time, to ES to achieve [^64^Cu][Cu(ES)]. The improved production method removed the need of a second synthesis module, as reported previously [27], and reduced the final volume of [^64^Cu]CuCl_2_ to 2 mL while maintaining similar yield and quality. In vitro studies in three cell lines from prostate cancer and glioblastoma showed that [^64^Cu][Cu(ES)] reduced cell survival more effectively than both [^64^Cu][Cu(ATSM)] and [^64^Cu]CuCl_2_. In vivo studies in xenografts in mice confirmed the superior therapeutic effects of [^64^Cu][Cu(ES)] and also demonstrated the ability of [^64^Cu][Cu(ES)] to detect tumor hypoxia using PET imaging.

Elesclomol was in the late 2000s investigated as a chemotherapeutic drug for treatment of metastatic melanoma, entering clinical trials both as a single agent and in combination with paclitaxel. However, ES failed short in a phase III clinical trial due to efficacy and toxicity concerns when used in combination with paclitaxel [19, 20, 30–33]. Recently, the interest in ES has regained, both for treating copper metabolism related disorders, and as a cancer treatment [34–39]. Notably, when used as a theranostic agent with Cu-64, the chemical amount of ES is much lower than when used as traditional chemotherapy. The mechanism of action of ES involves the transportation of copper into the mitochondria of tumor cells, targeting the mitochondrial electron transport chain (ETC), where Cu(II) is reduced to Cu(I), generating excess ROS and elevating oxidative stress, ultimately inducing cellular apoptosis [19–21, 30]. Recent investigations have suggested that cuproptosis, a newly discovered form of programmed cell death which involves copper binding to lipoylated components of the tricarboxylic acid cycle (TCA), might explain the mechanism of action of ES [39, 40].

One of the most important cellular responses to hypoxia is the activation of HIF-1α [41–44]. Under experimentally controlled hypoxia where the induction of HIF-1α was confirmed, we showed in our in vitro investigation that [^64^Cu][Cu(ES)] accumulates in tumor cells, and that the uptake of Cu-64 significantly increases compared to normoxic tumor cells treated with [^64^Cu][Cu(ES)] (Figs. 2 and 3). Among a series of adaptive genetic responses, HIF-1 regulates the mitochondrial respiration [45]. In hypoxia, HIF-1 actively downregulates oxygen consumption by inhibiting mitochondrial respiration, inducing the expression of pyruvate dehydrogenase kinase 1 (PDK1), inactivating the TCA cycle and diverting glucose metabolites to glycolysis, resulting in an altered ETC in mitochondria [42, 45–49]. Based on the mechanism of actions of ES, [^64^Cu][Cu(ES)] targets the mitochondrial metabolism in tumor cells and the increased accumulation in hypoxia is likely attributable to the altered ETC in dysfunctional mitochondria within hypoxic cells. The lack of oxygen as terminal electron acceptors in the mitochondrial respiratory chain produces a reductive intracellular microenvironment with excess nicotinamide adenine dinucleotide (NADH) and flavin adenine dinucleotide (FADH2), in which Cu(II) can be reduced to Cu(I) and retain in the region [19, 20, 30, 47, 50, 51]. This indicates that [^64^Cu][Cu(ES)] targets a dysfunctional, reductive mitochondrial microenvironment as a result of hypoxia.

Previously, [^64^Cu][Cu(ATSM)] was considered a promising theranostic candidate for hypoxic tumors [12–14, 52–54]. However, challenges and controversies regarding the retention mechanism of [^64^Cu][Cu(ATSM)] has limited its use, especially as similar results have been obtained for ionic Cu-64 substances, such as [^64^Cu]CuCl_2_ and ^64^Cu-acetate [10, 11, 55]. Our in vitro and in vivo studies demonstrated that [^64^Cu][Cu(ES)] reduced tumor cell survival and tumor growth significantly more effective than both [^64^Cu]CuCl_2_ and [^64^Cu][Cu(ATSM)] (Figs. 2 and 6). The lower therapeutic effects of [^64^Cu]CuCl_2_ is explained by that the ionic Cu(II) lacks passive penetration through the cellular membrane and relies solely on active copper transporter 1 (CTR-1) transportation, resulting in less cellular uptake and internalization. On the contrary, [^64^Cu][Cu(ATSM)], which has similar uptake and internalization in hypoxia as [^64^Cu][Cu(ES)], shows intriguing results. The reported hypoxic retention mechanism of [^64^Cu][Cu(ATSM)] involves the reduction of Cu(II) to Cu(I) through disturbed electron flow by NADH/NADPH in the dysfunctional mitochondria under hypoxia. This serves as an indicator of an overreduced intracellular state and mitochondrial disorder, which, to a certain extent, is comparable to the retention mechanism of [^64^Cu][Cu(ES)] [23, 56]. Other studies have challenged the proposed retention mechanism of [^64^Cu][Cu(ATSM)], highlighting the similarity in uptake and in vivo biodistribution between [^64^Cu][Cu(ATSM)] and the ionic compounds ^64^Cu-acetate or [^64^Cu]CuCl_2_, suggesting the instability of [^64^Cu][Cu(ATSM)] in blood and the potential role of copper itself in the retention mechanism [10, 11, 55]. Despite the controversies, our data suggests that [^64^Cu][Cu(ES)], [^64^Cu][Cu(ATSM)], and [^64^Cu]CuCl_2_ have different in vivo uptake in mice. This is seen in the tumor uptake (Fig. 4), but more prominently by the differences in brain uptake, where [^64^Cu]CuCl_2_ does not show any uptake, [^64^Cu][Cu(ATSM)] shows a unique hotspot uptake in the frontal region of the brain, and [^64^Cu][Cu(ES)] shows an overall uptake covering the entire brain (Fig. 5). This is an unexpected, yet very exciting finding that warrants further investigations and that might represent an avenue for developing a new treatment option for brain tumors. This result contradicts the hypothesis that [^64^Cu][Cu(ATSM)] disintegrates immediately upon contact with blood and that the uptake is solely through the copper metabolism pathways, but rather highlights the functional aspect of the complexing ligands. Considering the structural differences between [^64^Cu][Cu(ES)] and [^64^Cu][Cu(ATSM)], the discrepancy in biodistribution and cytotoxicity, despite the similarity of uptake mechanisms, may attribute to the difference in lipophilicity (logP = 2.45 for [^64^Cu][Cu(ES)] vs logP = 1.48 for [^64^Cu][Cu(ATSM)]), which might contribute to better tissue penetration through passive diffusion. The reduction potential (E° =  − 333 mV for [^64^Cu][Cu(ES)] vs E° =  − 590 mV for [^64^Cu][Cu(ATSM)]) of the copper complexes, which determines the point of reduction, is higher (less negative) for [^64^Cu][Cu(ES)] than [^64^Cu][Cu(ATSM)]), thus requires the TME to be more reductive [15, 20, 57, 58]. Meanwhile, Auger electron emission is the main cause of cytotoxicity of Cu-64, and the important aspect for Auger electrons to be effective is the close proximity to the target [49]. For Cu-64, the Auger electrons have an average energy of 2 keV and an average range of 126 nm with an estimated LET of 7 keV/μm [24, 25, 59]. The average size of a mammalian cell is approximately 10–100 μum in diameter, and a typical mitochondrion has a diameter of 0.5–1 μm [60, 61]. It may be hypothesized that [^64^Cu][Cu(ES)] is able to deliver Cu-64 into regions that are closer to nuclear and/or mitochondrial DNA, where the TME is more reductive, and even with a small decrease in distance, it might induce considerably more DNA double strand breaks (DSB) than [^64^Cu][Cu(ATSM)]. In addition, the effect of radiation from Cu-64 complements and amplifies the mechanism of action of ES, in that ES acts both as a vehicle to deliver Cu-64 into the sensitive region and by itself an active ROS generator. The possible synergetic effects of Cu-64, as a positron and Auger electron emitter, and ES, which targets the hypoxic dysfunctional mitochondrial metabolism, suggest that [^64^Cu][Cu(ES)] may be an ideal candidate for a hypoxia theranostic agent. Furthermore, by targeting the hypoxic mitochondria metabolism, [^64^Cu][Cu(ES)] may be effective against cancer stem cells (CSCs), in which mitochondria play an essential role. CSCs are proposed to selectively accumulate in regions where the TME is highly reductive while activation of HIF-1 promotes the epithelial-to-mesenchymal transition, although further investigations are needed to provide sufficient evidence [16–18, 62–67].

In our in vivo therapy study, we demonstrate the effect of [^64^Cu][Cu(ES)] through inhibited tumor growth in the 22Rv1 prostate cancer xenograft, in comparison with [^64^Cu][Cu(ATSM)] and untreated tumors (Fig. 6). Therapeutic effects of [^64^Cu]CuCl_2_ and [^64^Cu][Cu(ATSM)] have been previously demonstrated by Ferrari et al. and Yoshii et al. [13, 14]. The administration of a single dose of [^64^Cu][Cu(ES)] resulted in a larger growth inhibition and longer survival than a single dose of [^64^Cu][Cu(ATSM)]. Furthermore, multiple-dose treatment with [^64^Cu][Cu(ES)] gave a further and significant inhibition of tumor growth, although the survival was slightly less than for the mice receiving single-dose [^64^Cu][Cu(ES)], which can be explained by larger initial tumor volumes at the start of treatment (longest diameter of ~ 8 mm for [^64^Cu][Cu(ES)] multiple dose group vs ~ 5 mm for the other groups). This was caused by logistics that prevented production of [^64^Cu][Cu(ES)] when tumors had diameters of ~ 5 mm in both groups. Nevertheless, the in vivo tumor growth curve is consistent with the in vitro clonogenic (Fig. 2), demonstrating the that [^64^Cu][Cu(ES)] is a candidate therapeutic agent.

Although Cu-64 radiopharmaceuticals represent a promising potential as a theranostic agent, it is not without limitations. For instance, while the gamma emission benefits the imaging aspect, it also carries the risk of giving a gamma emission burden when a therapeutic dose is given. Therefore, continued investigations must be carried out to evaluate the benefits of using Cu-64 compared to other radionuclides, such as Cu-67. Although the brain uptake is an intriguing finding, this might limit the use of [^64^Cu][Cu(ES)] as a therapeutic agent in certain tumors. As almost all other radiocopper-based radiopharmaceuticals, Cu-64 is eventually accumulated in the liver, and thus results in a high liver dose. The implication, however, remains not well studied and would be of interest for further investigations.

## Conclusions

Following implementation of an improved production method of [^64^Cu]CuCl_2_, this is, to the best of our knowledge, the first time that ES is radiolabeled with [^64^Cu]CuCl_2_ to [^64^Cu][Cu(ES)]. In vitro and in vivo studies demonstrated that [^64^Cu][Cu(ES)] reduced cell survival and inhibited tumor growth more effectively than both [^64^Cu][Cu(ATSM)] and [^64^Cu]CuCl_2_. In addition, we demonstrated that PET imaging of tumor hypoxia with [^64^Cu][Cu(ES)] was feasible. Together, this shows the potential of [^64^Cu][Cu(ES)] as a promising theranostic agent for hypoxic solid tumors. The unexpected finding of brain uptake from [^64^Cu][Cu(ES)] is interesting and warrants further investigations.

### Supplementary Information

Below is the link to the electronic supplementary material.

Supplementary file1 (DOCX 29 KB)

## Data Availability

The datasets generated in the current study are available from the corresponding author on reasonable request.
