# Supplementary material for: In vitro and in vivo characterization of [64Cu][Cu(elesclomol)] as a novel theranostic agent for hypoxic solid tumors
Source: Eur J Nucl Med Mol Imaging. 2023 Jun 29;50(12):3576–88. doi: 10.1007/s00259-023-06310-4 (PMC10547809; doi:10.1007/s00259-023-06310-4)
Supplement: Supplementary file 1 — Supplementary file1 (DOCX 29 KB) [file 259_2023_6310_MOESM1_ESM.docx]

**Supplementary Information**

Title*: In vitro* and *in vivo* characterization of [^64^Cu][Cu(elesclomol)] as a novel theranostic agent for hypoxic solid tumors

Authors: Tengzhi Liu^1,2^, Maria Aanesland Dahle^1^, Mathilde Hirsum Lystad^1^, Laure Marignol^3^, Morten Karlsen^2,*^, Kathrine Røe Redalen^1,*^

Affiliations: ^1^Department of Physics, Norwegian University of Science and Technology, Trondheim, Norway; ^2^Department of Radiology and Nuclear Medicine, St. Olavs hospital, Trondheim University Hospital, Trondheim, Norway, ^3^Applied Radiation Therapy Trinity, Trinity St. James's Cancer Institute, Discipline of Radiation Therapy, Trinity College, Dublin, Ireland

*Shared last authors

Corresponding author: Kathrine Røe Redalen

Email of corresponding author: kathrine.redalen@ntnu.no

**Supplementary Methods**

***Synthesis of ATSM and ES***

The complex precursor for [^64^Cu][Cu(ATSM)], diacetyl-2-(4-N-methyl-3-thiosemicarbazone)-3-(4-N-amino-3-thiosemicarbazone) (H_2_ATSM) was synthesized according to a method based on previous reports [1,2]. The production of H_2_ATSM was confirmed using our previously described procedures [3]. 1,3-Bis[2-methyl-2-(phenylthioxomethyl)hydrazide]propanedioic acid (H_2_ES) was synthesized by a two-step synthesis from S-(thiobenzoyl)thioglycolic acid (99 %, Sigma-Aldrich, Burlington, MA, USA) [4] to ensure metal free precursor of good quality for radiolabeling. After the last step, 1.66 g of product was recrystallized from ether/pentane (35 mL) to yield white crystals of pure H_2_-ES in 88 % yield, ≥98 % purity, HPLC). ^1^H NMR (DMSO, 600 MHz): δ 7.30–7.39 (m, 5H), 6.86 (s, 2H), 3.26 (s, 3H); ^13^C NMR (DMSO): δ 182.3, 141.1, 128.4, 128.1, 126.2, 41.8.

***Synthesis of [^64^Cu]CuCl_2_, [^64^Cu][Cu(ATSM)] and [^64^Cu][Cu(ES)]***

Dissolution of the irradiated target was performed with 6 M HCl and passed through 1 mL tributyl phosphate (TBP, Triskem Brittany, France) and 2 mL TK201 resin (tertiary-amine-based weak ionic exchange resin, Triskem Brittany) columns in series. Ni-64 was recovered by several washes of 6 M HCl (6 mL) and collected. 4.5 M HCl was mixed in the modules syringe from 6 M and 0.2 M HCl and used to elute cobalt impurities (^55,56,57,58,61^Co) from the TK201 column (5 mL) [5]. The Cu-64 could then be eluted in a small volume of 0.2 M HCl (3 mL) to the reactor and evaporated to dryness. The evaporated HCl and water was trapped on a mixed molecular sieve and sodalime trap (250 mL). The dry product was reconstituted in the reactor from 0.2 M sodium acetate buffer at pH = 4.7 (3 mL) and transferred to a product vial at a typical volume activity of 2 GBq/ml. The radiosynthesis of [^64^Cu][Cu(ATSM)] and [^64^Cu][Cu(ES)] was performed from this solution directly by adding 15 μL of 1 mg/mL solution of the free chelator in DMSO to the buffered radioactive solution with 10% DMSO and a final volume of 0.5 mL. Synthesis was performed at room temperature for 10 min and yielded the product in quantitative yield. Radio-stability, molar activity, specific activity and radiochemical purity of [^64^Cu][Cu(ATSM)] and [^64^Cu][Cu(ES)] was measured with high performance liquid chromatography (HPLC) equipped with a radiodetector (Flow-RAM Radio-HPLC Detector, Lablogic), thin layer chromatography (TLC) and inductively coupled plasma mass spectroscopy (ICP-MS) after synthesis as previously described [3].

***Stability of [^64^Cu][Cu(ES)] in physiologic media***

20 µL of a 100 µM Cu(ES) solution was pipetted to 180 µL of mouse and human serum before incubation for 1, 4 and 24 hours, together with blank serum and saline spiked with 20 uL of a 100 uM Cu(ES) solution used as control. Samples were diluted with methanol (200 uL) and centrifuged. The supernatant was run on a high-performing liquid chromatography using a C18 rp18 column (320 nm). The area plotted against an external calibration curve was used to confirm linearity in the measurements.

***Cell lysates***

Cell lysates were prepared for analysis of uptake of radiopharmaceuticals. Whole cell lysates were extracted from 22Rv1 and PC3 cells treated with [^64^Cu]CuCl_2_, [^64^Cu][Cu(ATSM)], [^64^Cu][Cu(ES)] or placebo under normoxic and hypoxic conditions. Four days prior to treatment, two million cells were seeded in TC75 cell culture flasks with 14 mL of growth medium. Four hours before treatment, the hypoxic samples were placed in hypoxia and returned to the incubator, while the normoxic samples remained in the incubator. The treatment dose of each radiopharmaceutical was 4 Bq/cell. After the treatment, the hypoxic samples were placed in a freshly generated hypoxic environment for 4 hours before returning to normoxic conditions. Lysates were prepared by removing the media and washing the cells with 10 mL of ice-cold phosphate-buffered saline (PBS) twice, before 4 mL of ice-cold PBS was added, and the cells were removed with a cell scraper. The suspension was centrifuged at 2500 rpm for 10 minutes at 4 °C, and the supernatant was removed from the cell pellet. Lysis buffer (150 uL) consisting of mammalian protein extraction reagent (M-PER) with Halt protease inhibitor (1%) and Halt phosphatase inhibitor (1%) were added, followed by resuspension of the cells into the lysis buffer with vortexing. The mixture was incubated on ice for 30 minutes, before centrifuged at 24000 rcf at 4 °C for 20 minutes. The whole cell lysates were collected and stored at -80 °C.

Nuclear and cytoplasmic cell lysates were prepared from 22Rv1, PC3, and U-87MG cells. The preparation procedure followed the manufacturer’s protocol (NE-PER Nuclear and Cytoplasmic Extraction Reagents, Thermo Scientific). The number of cells seeded, the treatment, the hypoxic conditions and the removal of cells from the flasks were identical as preparation of whole cell lysates. The cell pellet was suspended in cytoplasmic extraction reagent I (CER I) before vortexed 15 seconds and incubated on ice for 10 minutes. Thereafter, the cytoplasmic extraction reagent II (CER II) was added followed by vortexing for 5 seconds. The cytoplasmic cell lysate was obtained as the supernatant after centrifuging the samples at 24000 rcf at 4°C for 5 minutes. The nuclear cell lysate was obtained from the pellet by adding nuclear extraction reagent (NER) and vortexing for 15 seconds. The samples were incubated on ice for 40 minutes while vortexed 15 seconds every 10 minute. Finally, samples were centrifuged at 24000 rcf for 10 minutes and the supernatant (nuclear lysate) was collected and stored at -80 °C.

***Western blotting***

Western blot (WB) was used to identify proteins of interest in the cell lysates. 10 μg of the protein lysate was pipetted into a 1.5 mL Eppendorf tube, along with 5 μL of NuPAGE LDS sample buffer (4X) (Thermo Fisher) and 2 μL of NuPAGE sample reducing agent (10X) (Thermo Fisher). The final volume of each sample was adjusted to 20 μL with Di-water, before heated to 80°C for 10 minutes to denature the proteins. Each sample was then loaded into a NuPAGE 10% Tris-Bis Mini Protein Gel (Thermo Fisher) placed into an XCell SureLock Mini Gel tank (Thermo Fisher) and filled with NuPAGE MES SDS running buffer. Two protein standards, the Precision Plus Protein Kaleidoscope and MagicMark XP Western Standard, were loaded on each gel along with the samples. Empty wells were filled with 5 μL of LDS buffer. The electrophoresis was initiated at 100 V for 30 minutes, followed by 150 V for 15 minutes and 200 V for 15-30 minutes until the load front approached the end of the gel. The gel was transferred to the XCell II Blotting module using transfer buffer and 30 V for one hour. The membrane was removed and rinsed with TBS buffer (with 0.2% tween-20, pH=7.5) twice, followed by washing with TBS-tween on a plate shaker at 4°C for 10 minutes twice. Detection of HIF-1α was performed with the purified mouse anti-human HIF-1α antibody clone 54 (BD Biosciences, Franklin Lakes, NJ, USA) as the primary antibody and horseradish peroxidase (HRP) goat anti-mouse antibody (BD Biosciences) as the secondary antibody. For loading control, rabbit monoclonal alpha-tubulin antibody (Abcam, Cambridge, UK) was used as the primary antibody and HRP goat anti-rabbit antibody (Novus Biologicals, Englewood, CO, USA) as the secondary antibody. The primary and secondary antibodies were diluted to a final volume of 10 mL with TBS-tween, based on the manufacturer’s recommendation (1:500 for HIF-1α and 1:1000 for the secondary antibody). The membrane was incubated in the primary antibody/TBS-tween solution with shaking at 4°C overnight before the membrane was washed three times with TBS-tween for 10 minutes and incubated with the secondary antibody for 1 hour at room temperature. Before imaging, the membrane was washed again for 10 minutes with TBS-tween.

***Cellular uptake and internalization***

Cellular whole cell uptake and internalization of Cu-64 radiopharmaceuticals in 22Rv1, PC3, and U-87MG cells under normoxic and hypoxic conditions were measured using a dose calibrator approximately 1.5 hours after the incubation. For whole cell uptakes, two million cells were seeded into a TC25 cell culture flask four days prior to treatment. Approximately three million PC3 and U87-MG cells, and four million 22Rv1 cells, were present in the flasks with upon treatment. Four hours before treatment, hypoxic samples were placed in the hypoxic condition. Subsequently, [^64^Cu]CuCl_2_, [^64^Cu][Cu(ATSM)] or [^64^Cu][Cu(ES)] were added at a dose of 4 Bq/cell. Hypoxic samples were returned to the hypoxic condition and normoxic samples were returned to the incubator. After four hours of incubation, the growth medium was transferred to 15 mL centrifuge tubes, and the radioactivity of the cells in the flasks and the mediums were measured. Whole cell uptake in Bq/cell was calculated using activity present in flasks divided by the number of cells in flasks. For hypoxic samples, the number of cells present in flasks after hypoxic incubation was estimated based on the clonogenic assay results. Internalization of Cu-64 radiopharmaceuticals was estimated from the nuclear and cytoplasmic cell lysates after measuring the activity of each sample. The protein concentration of each lysate was determined by bicinchoninic acid assay (BCA). The internalization was calculated using the non-decay corrected activity of each sample divided by the amount of protein as Bq/μg.

***Histology***

Tumor hypoxia was detected using a peroxidase-based immunostaining method, as previously described [6]. In brief, tissue sections were stained using the Dako EnVision™+ System-HRP (DAB) (K4011) and Dako autostainer. Deparaffinization and unmasking of epitopes were performed using PT-Link and EnVision™ Flex target retrieval solution with high pH. Sections were treated with 0.03% hydrogen peroxide for 5 minutes to block endogenous peroxidase. Incubation for 30 minutes with polyclonal rabbit antibodies to pimonidazole-protein adducts (1:10000 dilution) was followed by incubation with peroxidase-labeled polymer conjugated to goat anti-rabbit secondary antibodies for 30 minutes. Tissue sections were stained for 10 minutes with 3'3-diaminobenzidine tetrachloride and counterstained with haematoxylin, dehydrated and mounted in Diatex.

**Supplementary Results**

The stability of [64Cu][Cu(ES)] in physiologic media was evaluated in both mouse and human serum, confirming that Cu(ES) is stable in serum during 4 hours, as shown in Supplementary Figure S1.

**Supplementary Figure S1**. Stability of Cu(ES) solution in mouse and human serum after 1, 4 and 24 hours. Saline spiked with Cu(ES) solution was used as a control.

**References**

1. Gingras BA, Suprunchuk T, Bayley CH. The preparation of some thiosemicarbazones and their copper complexes: part III. Can J Chem. 1962;40:1053–9.

2. Christlieb M, Struthers HSR, Bonnitcha PD, Cowley AR, Dilworth JR. The exocyclic functionalisation of bis(thiosemicarbazonate) complexes of zinc and copper: the synthesis of monomeric and dimeric species. Dalton Trans. 2007;5043.

3. Liu T, Redalen KR, Karlsen M. Development of an automated production process of [^64^Cu][Cu(ATSM)] for positron emission tomography imaging and theranostic applications. J Labelled Comp Radiopharmac. 2022;65:191–202.

4. Yadav AA, Patel D, Wu X, Hasinoff BB. Molecular mechanisms of the biological activity of the anticancer drug elesclomol and its complexes with Cu(II), Ni(II) and Pt(II). J Inorg Biochem. 2013;126:1–6.

5. Jauregui-Osoro M, De Robertis S, Halsted P, Gould S-M, Yu Z, Paul RL, et al. Production of copper-64 using a hospital cyclotron: targetry, purification and quality analysis. Nucl Med Commun. 2021;42:1024–38.

6. Hagtvet E, Røe K, Olsen DR. Liposomal doxorubicin improves radiotherapy response in hypoxic prostate cancer xenografts. Radiat Oncol. 2011;6:135.
